# EHMTI-0229. A case of migraine like headache with postprandial hypoglycemia treated with lifestyle changing

**DOI:** 10.1186/1129-2377-15-S1-G39

**Published:** 2014-09-18

**Authors:** F Un Candan

**Affiliations:** 1 Neurology, Balikesir Public hospital, Balikesir, Turkey

## Introduction

Postprandial hypoglycemia(PH), is describing recurrent episodes of symptomatic hypoglycemia occurring after high carbonhydrated meal in people who don’t have diabetes. Hypoglycemia symptoms:blurry vision, fatigue, dizziness,sweating,headaches,numbness/coldness,confusion, coma.

The diagnosis was based upon reproduction of the patient's hypoglycemia symptoms in association with a blood glucose value of <70 mg/dL after an oral glucose tolerance test(OGTT). Fasting plasma glucose is less than 100 mg/dl.

## Case

The patient was healthy 32-year-old woman with repeated episodes of postprandial unilateral, throbbing headaches with nausea, vomitting, worsened by light for one year. She felt hungry before headache. She had to take more ten days NSAID per month.

Neurological examination, CT-MRI of the cranium were unremarkable. Blood tests, HbA1c was normal. Fasting plasma glucose was 74 mg/dl. In OGTT; 2 hour glucose was detected 64 mg/dl. Based on her clinical features and blood test, the patient was diagnosed with migraine-like headache secondary to PH.

She refused preventive therapy and began to use domperidon, naproksen and frovatriptan.And she changed lifestyle-nutrition; eating small meals-snacks about every 3 hours, avoiding sugar,exercising regularly, eating a variety of foods, choosing high-fiber.

During follow-up first month she had six episode of headache and took 6 times domperidon-naproksen and 3 times frovatriptan . In second month she took 4 times domperidon-naproksen, didn’t take frovatriptan. In third month she took 1 time domperidon-naproksen, didn’t take frovatriptan.

## Conclusion

We report this case to point to the physician to ask the patient with headache the relation of meal; also to show changing lifestyle and nutrition habit will resolve the headache instead of medications.

